# Selective Variceal Decompression: Current Status

**DOI:** 10.1155/1991/19213

**Published:** 1991

**Authors:** Gongliang Jin, Layton F. Rikkers

**Affiliations:** Department of Surgery University of Nebraska Medical Center 600 South 42nd Street Omaha Nebraska 68198-3280 USA

## Abstract

Since its introduction into clinical practice in 1967, selective variceal decompression by means of a distal splenorenal shunt (DSRS) has become one of the more commonly performed portal-systemic shunting procedures in the treatment of variceal hemorrhage throughout the world. In addition to selective decompression of gastroesophageal varices, the DSRS provides the advantages of preservation of portal perfusion of the liver and maintenance of intestinal venous hypertension. Many large, uncontrolled series and the majority of controlled randomized studies have demonstrated a lower incidence of encephalopathy after the DSRS than after nonselective shunt procedures. A secondary advantage of the DSRS is that the hepatic hilum is avoided, thus making subsequent liver transplantation a less formidable procedure. None of the studies have shown an advantage to this shunt with respect to longterm survival in patients with alcoholic cirrhosis. However, some of the large, uncontrolled series have shown that survival is significantly improved in patients with non-alcoholic cirrhosis compared to nonselective shunt procedures in the same population. Controlled trials comparing the DSRS to endoscopic sclerotherapy have shown that chronic endoscopic variceal sclerosis is an appropriate initial therapy for most patients as long as shunt surgery is readily available if sclerotherapy fails.


*HPB Surgery*, 1991, Vol. 5, pp. 1–15
Reprints available directly from the publisher
Photocopying permitted by license only

© 1991 Harwood Academic Publishers GmbH
Printed in the United Kingdom

### SELECTIVE VARICEAL DECOMPRESSION: CURRENT STATUS

GONGLIANG JIN and LAYTON F. RIKKERS
*Department of Surgery, University of Nebraska Medical Center, 600 South 42nd Street, Omaha, Nebraska 68198–3280, USA*

*(Received 30 May 1991)*

Since its introduction into clinical practice in 1967, selective variceal decompression by means of a distal splenorenal shunt (DSRS) has become one of the more commonly performed portal-systemic shunting procedures in the treatment of variceal hemorrhage throughout the world. In addition to selective decompression of gastroesophageal varices, the DSRS provides the advantages of preservation of portal perfusion of the liver and maintenance of intestinal venous hypertension. Many large, uncontrolled series and the majority of controlled randomized studies have demonstrated a lower incidence of encephalopathy after the DSRS than after nonselective shunt procedures. A secondary advantage of the DSRS is that the hepatic hilum is avoided, thus making subsequent liver transplantation a less formidable procedure. None of the studies have shown an advantage to this shunt with respect to long-term survival in patients with alcoholic cirrhosis. However, some of the large, uncontrolled series have shown that survival is significantly improved in patients with non-alcoholic cirrhosis compared to nonselective shunt procedures in the same population. Controlled trials comparing the DSRS to endoscopic sclerotherapy have shown that chronic endoscopic variceal sclerosis is an appropriate initial therapy for most patients as long as shunt surgery is readily available if sclerotherapy fails.

KEY WORDS: DSRS, Decompression, portal flow diversion

### RATIONALE AND HISTORY/BACKGROUND

Although surgical portal flow diversion was initially achieved by Nicolai Eck in 1877, Pavlov was the first to describe the physiologic consequences of the portaprival state[1]. In 1893, Pavlov's careful observations of dogs with portacaval shunts resulted in the description of the syndrome of "meat intoxication" or portasystemic encephalopathy. In addition, autopsy studies disclosed that animals that developed portasystemic encephalopathy had patent portacaval shunts and atrophic livers, whereas dogs with thrombosed or stenosed shunts had normal livers and hepatopetal portal collaterals. Pavlov concluded from these experiments that portal blood flow was essential for maintenance of liver function and structure and that complete portal diversion caused psychoneurologic dysfunction secondary to hepatic functional deterioration from reduced nutrient liver blood flow and to intestinally absorbed cerebral toxins bypassing hepatic detoxification.

Because there was no effective therapy for treatment of life-threatening variceal bleeding, Whipple and his associates introduced portacaval and conventional

Address correspondence to: Layton F. Rikkers, M.D., Department of Surgery, University of Nebraska Medical Center, 600 South 42nd Street, Omaha, Nebraska 68198–3280, USA

splenorenal shunts into clinical practice in 1945[2,3]. Their initial reports as well as several subsequent non-randomized studies showed that total portal decompression was an effective treatment for variceal hemorrhage. It was not until the 1960s, however, that the portacaval shunt was evaluated in randomized controlled trials[4]. Several such studies of both prophylactic and therapeutic portacaval shunts versus conventional medical treatment showed similar results: effective control of variceal hemorrhage, no survival advantage for patients with portacaval shunts, and accelerated hepatic failure and severe encephalopathy in patients with shunts. Although the portacaval shunt prevented hemorrhage, it did not prolong survival. It simply changed the mode of death from hemorrhage to hepatic failure.

These disappointing clinical results as well as animal studies, which demonstrated that portal blood contains hepatotrophic factors that are essential for liver maintenance and regeneration, were the major stimuli to search for a shunt which could control variceal hemorrhage but also maintain portal perfusion of the liver. The concept of selective variceal decompression by means of a distal splenorenal shunt (DSRS) was described in 1967 by Warren, Zeppa, and Fomon[5]. The operation was designed to provide selective transsplenic decompression of gastroesophageal varices, while preserving portal perfusion of the liver and maintaining mesenteric venous hypertension. They demonstrated that these objectives could be achieved by separating the splanchnic venous system into two compartments, a high pressure portomesenteric compartment and a low pressure gastrosplenic compartment, which is decompressed via a DSRS. These two components of the portal venous system were separated by interrupting the veins which connected them, including right and left gastric veins and the right gastroepiploic vein.

In 1968, Inokuchi described another selective shunt, the left gastric vena caval shunt[6]. This procedure achieves selective decompression of esophagogastric varices by anastomosis of the coronary vein to the inferior vena cava or interposition of a graft between these two veins. Even though this selective shunt was introduced at about the same time as the DSRS, experience with it has been almost entirely confined to Japan.

### DISTAL SPLENORENAL SHUNT

### *Indications and Contraindications*

An absolute contraindication to the DSRS is incompatible anatomy. In patients with prior splenectomy or splenic vein thrombosis, the DSRS is technically not feasible. In addition, we generally select another therapy for individuals whose splenic vein is less than 8 mm in diameter, because of the high incidence of shunt thrombosis in such patients.

Preoperative ascites has often been cited as a contraindication to the DSRS, because this procedure tends to aggravate rather than resolve ascites. However, most surgeons believe that transient preoperative ascites is not a contraindication to the DSRS. Ascites often develops after resuscitation from a variceal hemorrhage, but conservative medical treatment usually results in disappearance or stabilization of the ascites. The DSRS can generally be safely performed in such patients. Successful DSRS has also been reported in patients with massive ascites[7]. The incidence of post-shunt ascites in various series ranges from 10 to 52%[8].

Medical treatment resolves ascites in approximately 95% of patients after the DSRS. Peritoneovenous shunts should be reserved only for patients whose ascites is truly refractory to medical treatment. In our experience of 124 patients, the incidence of ascites severe enough to prolong hospitalization after the DSRS was 19% and only 2 patients (1.6%) required peritoneovenous shunting. Since severe postoperative ascites was a significant contributing factor to 10 postoperative deaths in our series, we prefer a side-to-side portal-systemic shunt (interposition or portacaval) rather than a DSRS for patients with variceal hemorrhage and medically intractable ascites.

In most centers, emergency surgery has played a decreasing role in the management of acute variceal hemorrhage as endoscopic sclerotherapy has become available. However, there remains an important role for emergency portasystemic shunting in patients who are refractory to nonoperative therapies[9,10,11]. The most frequently used emergency shunts are the end-to-side portacaval shunt and the interposition mesocaval shunt. Whether the DSRS should be used in the emergency setting is controversial. Many believe that the technical complexity of the procedure and the short-term incomplete variceal decompression provided by the DSRS are not suited to the emergency situation. However, when surgery is urgent rather than emergent (e.g. bleeding temporarily controlled by balloon tamponade), we believe that a DSRS is an effective and reasonable alternative. If the surgeon is experienced in performing this procedure, the operative mortality rate appears to be similar to other emergency operations. The experience with emergency DSRS has shown effective control of variceal bleeding in 95% of patients with mortality being related to the status of the underlying liver disease[12]. In a collected group of 173 patients undergoing emergency DSRS, the operative mortality rates ranged from 13 to 50% with an overall rate of 29%[7,10,12–19]. When possible, visceral angiography should precede surgery in patients undergoing the DSRS. In the emergency setting, the alternative of a distal splenocaval shunt should be considered, particularly when the left renal vein is abnormal or small[20,21]. An advantage of the distal splenocaval shunt is that it eliminates the possibility of postoperative renal venous hypertension[22].

In most Western centers, visceral angiography is done prior to the DSRS and this procedure is reserved for patients with hepatopetal portal flow. However, when medically intractable ascites is not a complicating factor, patients with absent hepatic portal perfusion may be reasonable candidates for this operation. We have found that portal flow to the liver is restored in some of these patients[23], and even in the absence of hepatic portal perfusion, the maintenance of intestinal venous hypertension after the DSRS may be beneficial, because it inhibits intestinal absorption of substances that may cause portasystemic encephalopathy[24].

Due to the limited resources in developing countries, it is impossible to perform routine angiography for every patient who undergoes the DSRS. In a collected group of 302 patients undergoing the DSRS, only 31 (10%) received preoperative angiographic studies. The results of DSRS in these patients were satisfactory with an overall operative mortality of 6%[7]. Thus, though desirable, preoperative angiography is not mandatory prior to a DSRS.

Most Chinese and Japanese surgeons advocate prophylactic shunts for patients with high risk varices which have not previously bled. Prophylactic shunts account for 25 to 39% of shunt operations in several Eastern series[7,25]. Of 302 DSRS reported in China, 102 (34%) were done prophylactically[7]. Of 231 patients receiving a left gastric-vena caval shunt, approximately 25% were prophylactic operations[25]. In a Japanese series of 78 patients,[19] 29 (37%) underwent a prophylactic DSRS, with an operative mortality of 3.4%; the 5-, 10- and 15-year survival rates all exceeded 85%. Despite these good results, no controlled trial has yet justified any shunt procedure for patients with varices which have not previously bled.

An additional reason to consider the DSRS is that dissection in the porta hepatis is avoided, thereby making the technical aspects of subsequent liver transplantation less difficult than if a portacaval shunt had been done[26]. Successful liver transplantation in patients with a previous DSRS has been reported[26-29]. Recently, Mazzaferro *et al.*[28] reported their experience of liver transplantation in 58 patients who had previous portasystemic shunts including 18 who had received the DSRS. They demonstrated that mortality after transplantation was less if the previous shunt procedure did not require liver hilum dissection. Complete portal vein thrombosis develops in 5% to 10% of patients after the DSRS. However, this complication does not eliminate the option of liver transplantation, which can be accomplished by interposing a vein graft between the donor portal vein and the recipient superior mesenteric vein[28].

### *Maintenance of Hepatic Portal Perfusion after the DSRS*

In addition to selective decompression of esophagogastric varices, two objectives of the DSRS are preservation of portal perfusion of the liver and maintenance of portal venous hypertension. Clinical results are best when both of these objectives are attained[30,31]. A major controversy regarding the DSRS is for how long and to what degree these variables are maintained postoperatively. Approximately 90% of patients with the DSRS have continuing portal flow in the early postoperative interval[32-34]. Loss of portal flow to the liver at this early point after the operation is most frequently secondary to portal vein thrombosis. Portal vein thrombosis as an early postoperative complication of the DSRS was first recognized in 1978[35]. Since then multiple reports have appeared with the incidence of total and partial portal vein thrombosis ranging from 5 to 19% and from 3 to 25%, respectively. The frequencies of total (10.5%) and partial portal vein thrombosis (17.7%) in our recent review of 124 patients with DSRS[36] are similar to those previously reported in the literature[33,37]. However, even when the portal vein is patent, the magnitude of portal flow is significantly decreased because of the loss of splenic venous flow. Analysis of 10 patients in our institution by duplex ultrasonography before and after the DSRS revealed diminution of portal flow by more than 50% in the early postoperative period[38].

A key factor in determining long-term maintenance of portal flow to the liver after the DSRS is etiology of liver disease. Patients with non-alcoholic cirrhosis and non-cirrhotic portal hypertension are more likely than patients with alcoholic cirrhosis to maintain hepatopetal portal flow in the late postoperative period[39]. Long-term follow-up of patients in the Emory randomized trial of DSRS versus nonselective shunt has shown that continuing portal flow to the liver was maintained for as long as 10 years in some patients with non-alcoholic cirrhosis[40]. In one series, 80% of non-alcoholic cirrhotics and only 25% of alcoholic cirrhotics had evidence of continuing portal perfusion of the liver at the one year evaluation[39]. The exact cause of this difference in post-shunt hemodynamic status between alcoholic and non-alcoholic cirrhosis is not clear. The group of patients most likely to maintain continuing hepatic portal perfusion after the DSRS are those with portal hypertension secondary to portal vein thrombosis[41] and to schistosomiasis,[42] but with normal liver architecture and function. Portal flow in patients with portal vein thrombosis is restored through hepatopetal collaterals, and since hepatic vascular resistance is normal, hepatic portal perfusion can usually be maintained indefinitely after the DSRS.

Other than the cause of portal hypertension, the extent to which the superior mesenteric venous part of the portal venous system is separated from the decompressed gastrosplenic component is another important factor in determining portal flow preservation. When the devascularization component of the operation is omitted, loss of portal flow through the coronary vein to the shunt is quite rapid[43]. However, even when portal-azygous disconnection is performed appropriately, portal flow is lost at a more rapid rate than in unshunted patients[44,45].

Recently both Warren[46] and Inokuchi[47] have shown that a major route for collateralization after the DSRS is through the pancreas. Since the splenic vein is in continuity with the pancreatic venous circulation via multiple small pancreatic branches, hepatic portal perfusion may gradually be lost through a “pancreatic siphon”[48]. To avoid the “pancreatic siphon” effect, both Warren[49] and Inokuchi[47] have suggested total disconnection of the splenic vein from the pancreas up to its bifurication at the splenic hilus, thereby separating the shunt from the pancreatic venous drainage. This extension of the DSRS operation is termed splenopancreatic disconnection. More extensive separation of the portomesenteric component of the splanchnic venous system from the decompressed gastrosplenic circuit is achieved with splenopancreatic disconnection[10,50–53]. In an initial evaluation of alcoholic cirrhotics at Emory, Warren and associates showed that portal perfusion of the liver at one year was preserved in a high percentage of patients who underwent splenopancreatic disconnection in addition to the DSRS, whereas few alcoholic cirrhotics had continuing hepatopetal flow to the liver when the standard DSRS was done. More recently, Henderson and co-workers[51] have reviewed the hemodynamic status of 78 patients undergoing DSRS and splenopancreatic disconnection. In their uncontrolled study, they found that hepatic portal perfusion was maintained in 84% of alcoholic and 90% of non-alcoholic patients at four years after surgery. After splenopancreatic disconnection, survival and late postoperative hepatic function were similar in alcoholic and non-alcoholic cirrhotics. Disadvantages of the procedure were longer operations, greater blood loss, and an increased incidence of recurrent hemorrhage from gastric varices[49]. Whether this variation on the DSRS operation will improve long-term clinical results has yet to be determined[11]. Since splenopancreatic disconnection is a more technically demanding operation than the originally described DSRS, we believe that it should not be recommended for routine application until its clinical benefits have been demonstrated in a randomized controlled trial. If performed by inexperienced surgeons, the splenic vein may be damaged, thus eliminating the option of selective variceal decompression.

Several studies have shown that there is gradual attrition of portal venous perfusion of the liver over subsequent months and years as collaterals from the high pressure portomesenteric venous compartment to the low pressure gastrosplenic system develop[54–56]. Data from Emory’s study showed that 98% of patients developed some collaterals at one year after the DSRS[48]. Alcoholic and nonalcoholic patients developed the same collateral pathways with pancreatic collaterals being most prominent in leading to gradual loss of portal flow in alcoholic patients[39,48]. However, despite the development of portal systemic collaterals, hepatic portal perfusion was preserved in 68 to 80% of patients after the DSRS[48,54].

Even when portal perfusion of the liver is markedly diminished or lost after the DSRS, maintenance of mesenteric venous hypertension may be beneficial by inhibiting intestinal absorption of nitrogenous substances that have been implicated in the pathogenesis of encephalopathy. We have shown that intestinal absorption of D-xylose is minimally changed one year after the DSRS, but significantly increased after nonselective shunts[24]. In addition, the higher postoperative blood ammonia concentrations in nonselective shunt patients correlated with the changes in D-xylose absorption induced by total portal decompression. Thus, even when portal flow is absent after the DSRS, residual portal hypertension may protect against the development of postoperative encephalopathy.

### *Non-Randomized Experience with the DSRS*

In 1984, Henderson *et al.* reviewed the world-wide non-randomized experience with the DSRS which had been accumulated over the prior 17 years[8]. The mean rates for operative mortality, shunt patency, and variceal rebleeding were 9%, 90%, and 7%, respectively. The reported incidences of encephalopathy ranged from 0 to 18%.

Since Henderson's report, the DSRS has become the shunt of choice for variceal hemorrhage in many centers. Series consisting of more than 2,700 patients undergoing the DSRS have been published in the English literature since 1984 (Table 1)[7,10,13–19,42,50,52,53,57–64]. This widespread application of the DSRS by surgical groups in North and Central America, Europe, Africa, and Asia is testimony to the successful adoption of this procedure by a large number of surgeons. In contrast to the randomized trials of the DSRS, which contain mainly patients with alcoholic cirrhosis, 70% of patients in these uncontrolled studies had non-alcoholic cirrhosis or noncirrhotic portal hypertension. In these series, 44% of patients were classified as Child's A, 42% as Child's B, 14% as Child's C. The results of the non-randomized studies were fairly consistent from series to series and identical with Henderson's previous report. This extensive uncontrolled experience with the DSRS cannot be meaningfully compared to most published series of nonselective shunts because the patient populations are so dissimilar. However, where comparisons can be made, the DSRS appears to be nearly as effective in preventing recurrent variceal hemorrhage and to be followed by a lower frequency of encephalopathy than nonselective shunts.

Between 1984 and 1990, 1,111 DSRS's were reported from 13 centers in Western countries[10,13,15–17,36,53,57–60,63,64]. These series ranged from 40 to 177 patients; the cause of portal hypertension was alcoholic cirrhosis in 50% of the patients. Since 1984, the Eastern experience (mainly Chinese and Japanese), encompasses large collected series with a combined total of 735 patients[7,19,50,52,61,62]. Prophylactic shunts accounted for 25 to 39% of shunt operations in several Eastern series. Over 90% of the patients had either posthepatitic or cryptogenic cirrhosis. The Western experience of the past seven years showed an overall operative mortality rate of 12%. This mortality rate, although mainly for elective operations, included an increasing

**Table 1** World-wide non-randomized experience with DSRS

| | *1967–1983* | *1984–1990*[b] | *Total* |
|---|---|---|---|
| Number of reports | 20 | 24 | 44 |
| Number of patients | >1,000 | >2,700 | >3,700 |
| Etiology: alcoholic cirrhosis (%) | 60 | 20 | 30 |
| nonalcoholic disease (%) | 40 | 80 | 70 |
| Child's Class: A (%) | 46 | 42[a] | 44 |
| B (%) | 40 | 43[a] | 42 |
| C (%) | 14 | 15[a] | 14 |
| Operative mortality (%) | 9 | 8 | 8 |
| Shunt patency (%) | 90 | 94 | >90 |
| Variceal rebleeding (%) | 7 | 6 | <7 |
| Encepalopathy (%) | 10 | 7 | <10 |
| 5-year survival (%) | 50–60 | 50–70 | 50–70 |

[a] Schistosomiasis patients not included
[b] Reports with patient number less than 40 not included number of emergency procedures in at least six reports[10,12–16]. Of 856 cases, 119 patients (14%) underwent an emergency operation. The operative mortality rates from the Eastern experience ranged from 1.8 to 15.8% with an overall rate of 5.7%. Decreasing operative mortality with increasing operative experience was reported in several studies[10,50,63]. The recurrent bleeding and shunt occlusion rates averaged approximately 7% in both Western and Eastern experiences. The overall incidence of all types of encephalopathy was 11.7% in Western reports and 6.5% in Eastern series. In most cases, encephalopathy was mild and could be reversed by conventional treatment. In most of these investigations, five-year survival rates were between 50% and 70%.

The two largest uncontrolled series from Western countries have demonstrated a survival advantage for patients with non-alcoholic cirrhosis compared with those with alcoholic cirrhosis[32,65]. However, several investigators have reported no significant difference in survival rates for alcoholic and non-alcoholic cirrhotics undergoing the DSRS[10,11,34,58,63,66]. It is not clear why the prognosis of non-alcoholic cirrhotics has varied among these different centers, but it probably relates to different types of non-alcoholic cirrhosis and to varying levels of hepatic functional reserve.

The most common cause of portal hypertension and esophagogastric variceal hemorrhage world-wide is hepatic schistosomiasis. Since the mid-1970s, groups in both the Middle East and South America have applied the DSRS to this population because of unacceptably high rates of encephalopathy after nonselective shunts and frequent recurrent bleeding in some series after devascularization operations. Since 1984, 887 schistosomiasis patients who have received a DSRS, have been reported[13,18,42,67,68]. The overall encephalopathy rate in these series is less than 2%, which is considerably less than the incidence of this complication after the DSRS in cirrhotic patients[40]. The five-year survival rate reported by Egyptian investigators in schistosomiasis patients after the DSRS was 91.5%, which was higher than survival figures in cirrhotic patients (75.6%) and mixed groups (65.2%) treated concomitantly in the same center[67]. Undoubtedly, these favorable results were obtained because liver function in the majority of patients was normal. It can be concluded from these series that the DSRS is an effective and safe procedure in the treatment of patients with schistosomiasis and variceal hemorrhage. Encephalopathy and hepatic failure after the DSRS are less frequent in schistosomal patients than in patients with alcoholic or non-alcoholic cirrhosis.

*Controlled Comparisons of the DSRS with Non-selective Shunts*

A total of seven randomized trials have compared the DSRS to a variety of nonselective shunts[40,69–74] (Table 2). In six of these studies, the patient populations of both groups were comprised of predominantly patients with alcoholic cirrhosis and the seventh trial contrasted the DSRS to a nonselective shunt and a non-shunting procedure in schistosomiasis patients. To date, no investigation has compared different shunt procedures in patients with non-alcoholic cirrhosis. A consistent finding in all of the studies is that survival was not improved after the DSRS.

Four of the seven trials[40,69,71,73] have shown a significantly lower frequency of postshunt encephalopathy in the DSRS patients; however, the other three trials have demonstrated no significant difference between selective and nonselective shunts in the incidence of this complication. In contrast to survival, encephalopathy is a subjective endpoint which was assessed with a variety of methods in the different trials. One of the more objective assessments quantified encephalopathy by the number of postoperative hospital admissions required for the treatment of this complication. This trial showed significantly fewer days of hospitalization for encephalopathy in the DSRS than in the portacaval shunt group[71]. These discrepancies in the frequencies of post-DSRS encephalopathy are probably due to differences in the patient populations studied, the methods used in assessing neuropsychological function and surgical technique. It is possible that technical variations resulting in less complete portal-azygous disconnection in the DSRS groups of two trials contributed to the higher incidence of post-DSRS encephalopathy observed in these studies[72,74].

Another important endpoint in comparing treatment for variceal hemorrhage is the effectiveness with which recurrent bleeding is prevented. Bleeding control was similar after selective and nonselective shunts in six of these studies[40,69–71,73,74] Likewise, selective and nonselective shunt occlusion rates were similar in these six trials. The Los Angeles study[72] showed a higher shunt failure rate after selective variceal decompression. The 30% incidence of recurrent variceal hemorrhage after the DSRS in that trial is the highest ever reported, suggesting a degree of surgeon inexperience with this procedure, as the frequency of DSRS failure was less than 10% for the other six studies combined.

In summary, these prospective randomized trials have shown no significant improvement in survival in alcoholic cirrhotic patients managed by the DSRS compared to those undergoing a nonselective shunt procedure. Selective variceal decompression provided a better quality of survival in four of the studies, even though three of these investigations contained mostly patients with alcoholic cirrhosis[75]. Prevention of recurrent variceal hemorrhage was equivalent for selective and nonselective shunts in six of the investigations. Although there are less controlled data, superiority of the DSRS may be more evident in patients with non-cirrhotic portal hypertension[68,73] and in those with non-alcoholic cirrhosis[32,65]. A single controlled trial in patients with schistosomiasis showed a lower frequency of

**Table 2** Randomized, controlled trials of DSRS versus nonselective shunts

| *Trial* | *Shunt* | *N* | *Alcoholic Cirrhosis(%)* | *Operative Mortality (%)* | *PSE (%)* | *Rebleeding (%)* | *Survival (%)* | *Follow-up (yr)* |
|---|---|---|---|---|---|---|---|---|
| Philadelphia (1979)[69] | DSRS | 13 | 100 | 8 | 0.75+* | NA | 69 | 3 |
| | MCS | 14 | 100 | 7 | 3.75+* | NA | 71 | 3 |
| Cincinnati (1981)[70] | DSRS | 23 | 96 | 4 | 5 | 13 | 78 | 2 |
| | SRS | 19 | 74 | 0 | 11 | 16 | 100 | 2 |
| Atlanta (1985)[40] | DSRS | 26 | 69 | 12 | 27* | 8 | 42 | 11 |
| | NSS | 29 | 76 | 10 | 76* | 14 | 28 | 11 |
| Toronto (1985)[71] | DSRS | 38 | 84 | 13 | 24* | 3 | 47 | 5 |
| | PCS | 40 | 80 | 0 | 40* | 0 | 53 | 5 |
| Los Angeles (1986)[72] | DSRS | 26 | 100 | 12 | 39 | 30* | 46 | 5 |
| | PCS | 27 | 100 | 7 | 32 | 4* | 33 | 5 |
| Sao Paolo (1986)[73] | DSRS | 30 | 0 | 0 | 7* | 7 | 100 | 2 |
| | SRS | 32 | 0 | 0 | 25* | 13 | 94 | 2 |
| New Haven/Boston (1988)[74] | DSRS | 43 | 88 | 9 | 51 | 18 | 53 | 3.5 |
| | NSS | 38 | 92 | 13 | 45 | 11 | 32 | 3.5 |

PSE = Portosystemic encephalopathy; DSRS = Distal splenorenal shunt; MCS = Mesocaval shunt; SRS = Conventional splenorenal shunt; NSS = Nonselective shunt; PCS = portacaval shunt; NA = Not available

* $P<0.05$

+ montsh PSE/months survival encephalopathy after DSRS than following a conventional splenorenal shunt. The morality rate was low after both procedures in this investigation.

*Controlled Comparisons of the DSRS with Endoscopic Sclerotherapy*

Four randomized controlled trials[44,45,76,77] have compared the DSRS to chronic sclerotherapy as definitive treatment for variceal hemorrhage in patients with cirrhosis (Table 3). Three of the studies[44,76,77] exclusively utilized the DSRS in the surgical group while the fourth trial,[45] conducted in our institution, utilized both selective (85%) and nonselective shunts (15%). All of the studies demonstrated significantly lower recurrent hemorrhage rates after the DSRS. In the two North American trials,[44,45] sclerotherapy failure secondary to uncontrolled recurrent variceal hemorrhage occurred in approximately one-third of patients. In the Atlanta trial,[44] 82% of sclerotherapy failures were salvaged by shunt surgery, whereas only 20% of sclerotherapy failures were salvaged in the Omaha study[45]. These contrasting salvage rates are most likely due to the different patient populations of the two trials. The Omaha study contained a higher percentage of patients with alcoholic cirrhosis, who lived in relatively remote geographic areas. Many of these patients died from recurrent hemorrhage before they could be transferred for shunt surgery. Sclerotherapy with surgical rescue in one-third of patients resulted in significantly longer survival than initial DSRS in the Atlanta trial. However, survival curves for sclerotherapy and shunt surgery were not significantly different in the Omaha study[45]. The investigations by Teres and co-workers in Spain and Spina and co-workers in Italy also found no significant difference in survival after the DSRS and endoscopic sclerotherapy, despite the higher rebleeding rate in the sclerotherapy group.

Three of these studies[44,45,76] showed no significant difference in frequency of encephalopathy after the DSRS and endoscopic sclerotherapy, while the fourth investigation[77] demonstrated a higher encephalopathy rate after the DSRS (24%) than after endoscopic variceal sclerosis (8%). This may have been due to the modified surgical technique used, which did not include portal-azygous disconnection with the DSRS. These investigators concluded that endoscopic sclerotherapy is a reasonable alternative to selective shunting for long-term management of variceal hemorrhage, especially in patients who are prone to encephalopathy.

Two of these studies evaluated the effect of the DSRS and endoscopic sclerotherapy on hepatic hemodynamics and quantitative liver function[44,45]. Significantly more patients had continuing hepatic portal perfusion at the one-year assessment in the sclerotherapy groups of both of these investigations. This difference in portal flow between treatment groups resulted in significantly better hepatic functional reserve in the sclerotherapy group of the Atlanta study but not in the Omaha trial.

Our conclusion from these four studies is that chronic sclerotherapy is an acceptable, but not superior, alternative to the DSRS for management of patients with variceal hemorrhage. Recurrent variceal hemorrhage is common after definitive sclerotherapy and this treatment modality will fail in approximately one-third of patients. Although sclerotherapy may be the preferred initial treatment for many variceal bleeders, patients living in remote areas or those who are non-compliant with respect to their sclerotherapy schedule should probably undergo an initial shunt operation rather than receive chronic sclerotherapy.

**Table 3** Controlled trials of endoscopic sclerotherapy versus DSRS

| | *Rikkers*[45] | *Warren*[44] | *Teres*[77] | *Spina*[76] |
|---|---|---|---|---|
| 2-Year survival after DSRS (%) | 65 | 59* | 71 | 95 |
| after EVS (%) | 61 | 84* | 68 | 90 |
| Encephalopathy after DSRS (%) | 16 | 16 | 24* | 5 |
| after EVS (%) | 7 | 12 | 8* | 10 |
| Rebleeding after DSRS (%) | 19* | 3* | 14* | 5* |
| after EVS (%) | 57* | 53* | 38* | 35* |

DSRS = Distal splenorenal shunt; EVS = Endoscopic variceal sclerotherapy
* $p<0.05$

### THE LEFT GASTRIC-VENA CAVAL SHUNT

The left gastric-vena caval shunt was proposed by Inokuchi in 1968[6]. In 1990, Inokuchi and associates[52] reported their experience with this operation in 258 patients with either cirrhosis or idiopathic portal hypertension. Approximately 25% of the operations were done prophylactically in patients who had never bled from varices. Postoperative encephalopathy was infrequent, recurrent variceal hemorrhage complicated the course of 8% of patients, and the shunt was patent in 88% of patients who underwent postoperative angiography. Although results with this procedure in patients with non-alcoholic cirrhosis have been excellent, the left gastric-vena caval shunt has not been compared to other therapies in a randomized, controlled trial and no large series of patients with alcoholic cirrhosis undergoing this procedure has been reported. This procedure has occasionally been used as an alternative means of selective shunting in patients in whom prior splenectomy has obviated a DSRS[46]. In many patients with portal hypertension, the coronary vein is thin and friable, which adds to the technical difficulty of the procedure. Furthermore, patients with alcoholic cirrhosis frequently have edematous, thickened retroperitoneal tissue which makes this operation more difficult to accomplish. Most likely because of these factors, this selective shunt has not been widely applied outside of Japan.

### CONCLUSIONS

The available data from randomized trials and the world-wide non-randomized experience have led us to the following conclusions regarding the current status of the DSRS:

1. The DSRS has gained widespread acceptance for patients with variceal hemorrhage with more than 2,700 cases being reported in the world literature since 1984.
2. The DSRS is nearly as effective as nonselective shunts in preventing recurrent hemorrhage.
3. The major contraindications to the DSRS are medically intractable ascites and anatomic limitations (prior splenectomy or splenic vein less than 8mm in diameter).

4. When surgery is urgent rather than emergent, a DSRS is an effective and reasonable alternative for many patients.
5. Hepatic portal perfusion after the DSRS can be maintained in the majority of patients during the early postoperative interval. However, in many patients (mainly alcoholic cirrhotics), portal flow gradually collateralizes to the shunt. Portal flow may be better preserved after splenopancreatic disconnection, but no clinical benefits have yet been established for this technically difficult extension of the DSRS.
6. Randomized trials of the DSRS versus nonselective shunts, which have included mainly patients with alcoholic cirrhosis, have shown no advantage to the DSRS with respect to long-term survival. Some large, nonrandomized experiences, have demonstrated longer survival in non-alcoholic than in alcoholic cirrhotics.
7. The majority of controlled trials and most nonrandomized comparisons have demonstrated a lower frequency of encephalopathy after the DSRS than after nonselective shunts. This difference is particularly striking in patients with schistosomiasis.
8. Chronic sclerotherapy and the DSRS have provided similar results in most controlled trials. In settings where sclerotherapy failure can be effectively salvaged by shunt surgery, chronic sclerotherapy is the preferable initial treatment. However, the DSRS may be the superior initial therapy for individuals living in remote geographic areas and for those who are likely to be noncompliant.

### *References*

1. Hahn, M., Massen, O., Nencki, M. and Pavlov, J. (1893) Die eck sche fistel zwischen der unteren hohevene and der pfortaden und ihre folgen fur den organismus. *Arch. Exp. Pathol. Pharmokol.*, **32**, 162–210
2. Whipple, A.O. (1945) The problem of portal hypertension in relation to the hepatosplenopathies. *Ann. Surg.*, **122**, 449–475
3. Blakemore, A.H. and Lord, J.W. Jr. (1945) The technic of using Vitalium's tubes in establishing portacaval shunts for portal hypertension. *Ann. Surg.*, **122**, 476–489
4. Rikkers, L.F. (1987) Bleeding esophageal varices. *Surg. Clin. North Am.*, **67**, 475–488
5. Warren, W.D., Zeppa, R. and Fomon, J.J. (1967) Selective transsplenic decompression of gastroesophageal varices by distal splenorenal shunt. *Ann. Surg.*, **166**, 437–455
6. Inokuchi, K. (1968) A selective portacaval shunt. *Lancet*, **2**, 51–52
7. Jin, G. (1990) Current status of distal splenorenal shunt in China. *Am. J. Surg.*, **160**, 93–97
8. Henderson, J.M., Millikan, W.J. and Warren, W.D. (1984) The distal splenorenal shunt: an update. *World. J. Surg.*, **8**, 722–732
9. Rikkers, L.F. (1989) Invited commentary: Emergency portasystemic shunting in cirrhotic with bleeding varices: a comparison of portacaval and mesocaval shunts. *HPB Surgery*, **1**, 116–118
10. Myburgh, J.A. (1990) Selective shunts: the Johannesburg experience. *Am. J. Surg.*, **160**, 67–74
11. Langer, B., Taylor, B.R. and Greig, P.D. (1990) Selective or total shunts for variceal bleeding. *Am. J. Surg.*, **160**, 75–79
12. Potts, J.R., Henderson, J.M., Millikan, W.J. Jr. and Warren, W.D. (1984) Emergency distal splenorenal shunts for variceal hemorrhage refractory to nonoperative control. *Am. J. Surg.*, **148**, 813–816
13. Orozco, M., Mercado, M.A., Takahashi, T., Garcia-Tsao, G., Guevara, L., Ortiz, H., Hernandez-Cendejas, A. and Tielve, M. (1990) Role of the distal splenorenal shunt in management of variceal bleeding in Latin America. *Am. J. Surg.*, **160**, 86–89

14. Lodge, J.P.A., Mavor, A.I.D. and Giles, G.R. (1989) Distal splenorenal versus lienorenal shunt for acute variceal hemorrhage: is the selective shunt an advance? *J. Roy. Coll. Surg. Edin.*, **34**, 59--62
15. Mitchell, R.L. and Ignatius, J.A. (1988) Distal splenorenal shunt: standard procedure for elective and emergency treatment of bleeding esophageal varices. *Am. J. Surg.*, **156**, 169–192
16. Pomerantz, R.A., Eckhauser, F.E., Knol, J.A., Guirre, K., Raper, S.E. and Turcotte, J.G. (1989) Operative timing and patient survival following distal splenorenal shunt. *Amer. Surg.*, **55**, 333–337
17. Busuttil, R.W. (1984) Selective and nonselective shunts for variceal bleeding. *Am. J. Surg.*, **148**, 27–35
18. Raia, S., Mies, S. and Macedo, A.L. (1984) Surgical treatment of portal hypertension in schistosomiasis. *World. J. Surg.*, **8**, 738–752
19. Nagasue, N., Kohno, H., Ogawa, Y., Yukaya, H., Tamada, R., Sasaki, Y., Chang, Y.C. and Nakamura, T. (1989) Appraisal of distal splenorenal shunt in the treatment of esophageal varices. An analysis of prophylactic, emergency and elective shunts. *World. J. Surg.*, **13**, 92–99
20. Iliopoulos, J.I., Thomas, J.H., Pierce, G.E. and Hermreck, A.S. (1988) Splenocaval shunt for selective portal decompression. *World. J. Surg.*, **12**, 852–854
21. Ross, C., Potts, J.R. III (1988) Early experience with the selective splenocaval shunt for variceal bleeding. *Surg. Gynecol. Obstet.*, **167**, 335–340
22. Rikkers, L.F. (1988) Invited commentary: Splenocaval shunt for selective portal decompression. *World J. Surg.*, **12**, 854–855
23. Rikkers, L.F. (1990) Portal hypertension in surgical treatment of digestive disease, 2nd edn, pp. 363–380. Chicago: Year Book Medical Publishers Press
24. Rikkers, L.F. (1983) Portal hemodynamics, intestinal absorption, and postshunt encephalopathy. *Surgery*, **94**, 126–133
25. Inokuchi, K., Beppu, K. and Koyanagi, N. (1984) Fifteen years' experience with left gastric venous caval shunt for esophageal varices. *World J. Surg.*, **8**, 716–721
26. Rikkers, L.F. (1989) Invited commentary: Liver transplantation in the treatment of bleeding esophageal varices. *HPB Surgery*, **1**, 366–369
27. Wood, R.P., Shaw, B.W., Rikkers, L.F. (1990) Liver transplantation for variceal hemorrhage. *Surg. Clin. North. Am.*, **70**, 449–462
28. Mazzaferro, V., Todo, S., Tzakis, A.G., Stieber, A.C., Makowka, L., Starzl, T.E. (1990) Liver transplantation in patients with previous portasystemic shunt. *Am. J. Surg.*, **160**, 111–116
29. Esquival, C.O., Klintmalm, G., Iwatsuki, S., Makowka, L., Gordon, R.D., Tzakis, A. and Starzl, T.E. (1987) Liver transplantation in patients with patent splenorenal shunts. *Surgery*, **207**, 430–432
30. Henderson, J.M. (1990) The distal splenorenal shunt. *Surg. Clin. North Am.*, **70**, 405–423
31. Rikkers, L.F., Cormier, R.A. and Vo, N.M. (1987) Effects of altered portal hemodynamics after distal splenorenal shunts. *Am. J. Surg.*, **153**, 80–83
32. Warren, W.D., Millikan, W.J. Jr., Henderson, J.M., Wright, L., Kutner, M., Smith, R.B. III, Fulenwide, J.T., Salam, A.A. and Galambos, J.T. (1982) Ten year's portal hypertensive surgery at Emory. *Ann. Surg.*, **195**, 530–542
33. Spina, G.P., Santambrogio, R., Opocher, E., Gattoni, F., Baldini, U., Cucchiaro, G., Uslenghi, C. and Pezzuol, G. (1990) Early hemodynamic changes following distal splenorenal shunt for portal hypertension. *World J. Surg.*, **14**, 115–122
34. Rikkers, L.F., Soper, N.J. and Cormier, R.A. (1984) Selective operative approach for variceal hemorrhage. *Am. J. Surg.*, **147**, 89–96
35. Rikkers, L.F., Rudman, D., Galambos, J.T., Fulenwider, T., Millikan, W.J., Kutner, M., Smith, R.B. III, Salam, A.A., Sones, P.J. and Warren, W.D. (1978) A randomized controlled trial of the distal splenorenal shunt. *Ann. Surg.*, **188**, 271–282
36. Jin, G. and Rikkers, L.F. (in press) Significance of portal vein thrombosis after distal splenorenal shunt. *Arch. Surg.*
37. Henderson, J.M., Millikan, W.J., Chipponi, J., Wright, L., Sones, P.J., Meier, L. and Warren, W.D. (1982) The incidence and natural history of thrombosis in the portal vein following distal splenorenal shunt. *Ann. Surg.*, **196**, 1–7
38. Ozaki, C.F., Anderson, J.C., Lieberman, R.P. and Rikkers, L.F. (1988) Duplex ultrasound as a noninvasive technique of assessing portal hemodynamics. *Am. J. Surg.*, **155**, 70–74
39. Henderson, J.M., Millikan, W.J., Wright-Bacon, L., Kutner, M.H. and Warren, W.D. (1983) Hemodynamic differences between alcoholic and nonalcoholic cirrhotics following distal splenorenal shunt: effect on survival? *Ann. Surg.*, **198**, 325–334

40. Millikan, W.J., Warren, W.D., Henderson, J.M., Smith, R.B. III, Salam, A.A., Galambos, J.T., Kutner, M.H. and Keen, J.H. (1985) The Emory prospective randomized trial: selective versus nonselective shunt to control variceal bleeding. *Ann. Surg.*, **201**, 712–722
41. Galloway, J.R. and Henderson, J.M. (1990) Management of variceal bleeding in patients with extrahepatic portal vein thrombosis. *Am. J. Surg.*, **160**, 122–127
42. Salam, A.A., Ezzat, F.A. and Abu-Elmagd, K.M. (1990) Selective in schistosomiasis in Egypt. *Am. J. Surg.*, **160**, 90–92
43. Vang, J., Simert, G., Hansson, J., Thylen, U. and Bengmark, S. (1977) Results of a modified distal splenorenal shunt for portal hypertension. *Ann. Surg.*, **185**, 224–228
44. Warren, W.D., Henderson, J.M., Millikan, W.J., Galambos, J.T., Brooks, W.S., Riepe, S.P., Salam, A.A. and Kutner, M.H. (1986) Distal splenorenal shunt versus endoscopic sclerotherapy for long-term management of variceal bleeding. Preliminary report of a prospective, randomized trial. *Ann. Surg.*, **203**, 454–462
45. Rikkers, L.F., Burnett, D.A., Volentine, G.D., Buchi, K.N. and Cormier, R.A. (1987) Shunt surgery versus endoscopic sclerotherapy for long-term treatment of variceal bleeding. *Ann. Surg.*, **206**, 261–271
46. Warren, W.D., Millikan, W.J. Jr., Henderson, J.M., Rasheed, M.E. and Salam, A.A. (1984) Selective variceal decompression after splenectomy or splenic vein thrombosis. *Ann. Surg.*, **199**, 694–702
47. Inokuchi, K., Beppu, K., Koyanagi, N., Nagamine, K., Hashizume, M. and Sugimachi, K. (1984) Exclusion of nonisolated splenic vein in distal splenorenal shunt for prevention of portal malcirculation. *Ann. Surg.*, **200**, 711–717
48. Henderson, J.M., Jin, G.L., Galloway, J., Millikan, W.J. Jr., Sones, P.J. and Warren, W.D. (1986) Portaprival collaterals following distal splenorenal shunt: incidence, magnitude, and associated portal perfusion changes. *J. Hepatol.*, **1**, 649–661
49. Warren, W.D., Millikan, W.J., Henderson, J.M., Abu-Elmagd, K.M., Galloway, J.R., Shires, G.T. III, Richards, W.O., Salam, A.A. and Kutner, M.H. (1986) Splenopancreatic disconnection: improved selectivity of distal splenorenal shunt. *Ann. Surg.*, **204**, 346–355
50. Ashida, H., Utsunomiya, J., Kotoura, Y., Ishikawa, Y., Nishioka, A., Takagi, K. and Fukuda, M. (1989) Results of distal splenorenal shunt with versus without splenopancreatic disconnection. *J. Clin. Gastroenterol*, **11**, 658–662
51. Henderson, J.M., Warren, W.D., Millikan, W.J., Galloway, J.R., Kawasaki, S. and Kutner, M.H. (1989) Distal splenorenal shunt with splenopancreatic disconnection: a 4-year assessment. *Ann. Surg.*, **210**, 332–340
52. Inokuchi, K. and Sugimachi, K. (1990) The selective shunt for variceal bleeding: a personal perspective. *Am. J. Surg.*, **160**, 48–53
53. Maffei-Faccioli, A., Gerunda, G.E., Neri, D., Merenda, R., Zangrandi, E. and Meduri, F. (1990) Selective variceal decompression and its role relative to other therapies. *Am. J. Surg.*, **160**, 60–68
54. Belghiti, J., Grenier, P., Nouel, O., Nahum, H. and Fekete, F. (1981) Long-term loss of Warren's shunt selectivity: angiographic demonstration. *Arch. Surg.*, **116**, 1121–1124
55. Maillard, J.N., Flamant, Y.M., Hay, J.M. and Chandler, J.G. (1979) Selectivity of the distal splenorenal shunt. *Surgery*, **86**, 663–671
56. Widrich, W.C., Robbins, A.H., Johnson, W.C. and Nabseth, D.C. (1980) Long-term follow-up of distal splenorenal shunts: evaluation by arteriography, shuntgraphy, transhepatic portal venography and cinefluorography. *Radiology*, **134**, 341–345
57. Rigau, J., Teres, J., Visa, J., Bosch, J., Consesa, A., Grande, L., Vilar, J.A., Garcia-Valdecasas J.C. and Pera, C. (1986) Long-term follow-up of 100 patients with portal hypertension treated by a modified splenorenal shunt. *Brit. J. Surg.*, **73**, 708–711
58. Adson, M.A., Van Heeden, J.A. and Ustrup, D.M. (1984) The distal splenorenal shunt. *Arch. Surg.*, **119**, 609–614
59. Shah, D.M., Chang, B.B., White, T.Z., Feustel, P.J., Kaufman, J.L. and Leather, R.P. (1989) Comparison between selective distal splenorenal shunt and small diameter H-graft portosystemic shunt. *J. Cardiovasc. Surg.*, **30**, 459–461
60. Peterson, K. and Giles, G.R. (1986) Distal spolenorenal (Warren) shunt in the management of actively bleeding esophageal varices. *Brit. J. Surg.*, **73**, 618–620
61. Isomatsu, T. (1988) Indication and results of distal splenorenal shunt. In Treatment of Esophageal Varices, edited by Y. Idezuki, pp. 287–298. Elsevier Science Publisher B.V.
62. Katoh, H. and Tanabe, T. (1988) Long-term results of super selective distal splenorenal shunt. In Treatment of Esophageal Varices, edited by Y. Idezuki, pp. 299–302. Elsevier Science Publisher B.V.

63. Pezzuoli, G., Spina, G., Santambrogio, R., Galeotti, F., Opocher, E., Cucchiaro, G., Lopez, C. and Strinna, M. (1987) The distal splenorenal shunt: an update on experience of 106 cases. *Int. Surg.*, **72**, 144–148
64. Saubier, E.G. and Gouillat, C. (1984) Results of the Warren operation for portal hypertension. *Ann. Gastroenterol Hepatol.*, **20**, 7–11
65. Zeppa, R., Hutson, D.G., Levi, J.U. and Levingstone, A.S. (1984) Factors influencing survival after distal splenorenal shunt. *World J. Surg.*, **8**, 733–737
66. Orozco, H., Juarez, F., Santillan, P., Gonzalez, O., Guevara, L., Hernandez, J., Mercado, M.A., Ordorica, J., Guraieb, E., Uribe, M. and Takahashi, T. (1988) Ten years of selective shunts for hemorrhagic portal hypertension. *Surgery*, **103**, 27–31
67. Ezzat, F.A., Abu-Elmagd, K.M., Sultan, A.A., Aly, M.A., Fathy, O.M., Bahgat, O.O., El-Fiky, A.M., El-Barbary, M.H. and Mashhoor, N. (1989) Schistosomal versus nonschistosomal variceal bleeders — do they respond differently to selective shunt (DSRS)? *Ann. Surg.*, **209**, 489–500
68. Ezzat, F.A., Abu-Elmagd, K.M., Aly, M.A., Fathy, O.M., El-Ghawlby, N.A., El-Fiky, A.M. and El-Barbary, M.H. (1990) Selective shunt versus nonshunt surgery for management of both schistosomal and nonschistosomal variceal bleeders. *Ann. Surg.*, **212**, 97–108
69. Reichle, F.A., Fahmy, W.F. and Golsorkhi, M. (1979) Prospective comparative clinical trial with distal splenorenal and mesocaval shunts. *Am. J. Surg.*, **137**, 13–21
70. Fisher, J.E., Bower, R.H., Atamian, S. and Welling, R. (1981) Comparison of distal and proximal splenorenal shunts: a randomized prospective trial. *Ann. Surg.*, **194**, 531–544
71. Langer, B., Taylor, B.R., Mackenzie, D.R., Gilas, T., Stone, R.M. and Blendis, L. (1985) Further report of a prospective randomized trial comparing distal splenorenal shunt with end-to-side portacaval shunt. *Gastroenterology*, **88**, 424–429
72. Harley, H.A., Morgan, T., Redeker, A.G., Reynolds, T.B., Villamil, F., Weiner, J.M. and Yellin, A. (1986) Results of a randomized trial of end-to-side portacaval shunt and distal splenorenal shunt in alcoholic liver disease and variceal bleeding. *Gastroenterology*, **91**, 802–809
73. da Silva, L.C., Strauss, E., Gayotto, L.C.C., Mies, S., Macedo, A.L.., da Silva, A.T., Silva, E.F., Lacet, C.M.C., Antonelli, R.H., Fermanian, J., Foster, S., Raia, A. and Raia, S. (1986) A randomized trial for the study of elective surgical treatment of portal hypertension in Mansonic schistosomiasis. *Ann. Surg.*, **204**, 148–153
74. Grace, D.N., Conn, H.O., Resnick, R.H., Groszmann, R.J., Atterbury, C.E., Wright, S.C., Gusberg, R.J., Vollman, R., Garcia-Tsao, G., Fisher, R.L., O'Hara, E.D., McDermott, W.V., Maselli, J.P., Widrich, W., Matloff, D.S., Horst, D., Banks, N. and Alberts, J. (1988) Distal splenorenal vs. portal-systemic shunts after hemorrhage from varices: a randomized controlled trial. *Hepatology*, **8**, 1475–1481
75. Rikkers, L.F. (1988) Is the distal splenorenal shunt better? *Hepatology*, **8**, 1705–1707
76. Spina, G.P., Santambrogia, R., Opocher, E., Cosentino, F., Zambelli, A., Passoni, G.R., Cucchiaro, G., Macri, M., Morandi, E., Bruno, S. and Pezzuoli, G. (1989) Distal splenorenal shunt versus endoscopic sclerotherapy in prevention of variceal rebleeding. *Ann. Surg.*, **211**, 178–186
77. Teres, J., Bordas, J.M., Bravo, D., Visa, J., Grande, L., Garcia-Valdecasas, J.C., Pera, C., Rodes, J. (1987) Sclerotherapy vs. distal splenorenal shunt in the elective treatment of variceal hemorrhage. A randomized controlled trial. *Hepatology*, **7**, 430–436

*(On invitation by S. Bengmark 21 May 1991)*

